# Adjunctive Application of Antimicrobial Photodynamic Therapy in Nonsurgical Periodontal Treatment: A Review of Literature

**DOI:** 10.3390/ijms161024111

**Published:** 2015-10-13

**Authors:** Takeshi Kikuchi, Makio Mogi, Iichiro Okabe, Kosuke Okada, Hisashi Goto, Yasuyuki Sasaki, Takeki Fujimura, Mitsuo Fukuda, Akio Mitani

**Affiliations:** 1 Department of Periodontology, School of Dentistry, Aichi Gakuin University, Nagoya, Aichi 464-8651, Japan; E-Mails: ag133d04@dpc.agu.ac.jp (I.O.); ag133d03@dpc.agu.ac.jp (K.O.); ag123d14@dpc.agu.ac.jp (H.G.); ag143d09@dpc.agu.ac.jp (Y.S.); takeki@dpc.agu.ac.jp (T.F.); fukuda-m@dpc.agu.ac.jp (M.F.); 2 Department of Medicinal Biochemistry, School of Pharmacy, Aichi Gakuin University, Nagoya, Aichi 464-8650, Japan; E-Mail: makio@dpc.agu.ac.jp

**Keywords:** photodynamic therapy, antimicrobial photodynamic therapy, periodontal treatment, periodontal disease, periodontal pathogenic bacteria, low-power laser, photosensitizer

## Abstract

Periodontal disease is caused by dental plaque biofilms, and the removal of these biofilms from the root surface of teeth plays a central part in its treatment. The conventional treatment for periodontal disease fails to remove periodontal infection in a subset of cases, such as those with complicated root morphology. Adjunctive antimicrobial photodynamic therapy (aPDT) has been proposed as an additional treatment for this infectious disease. Many periodontal pathogenic bacteria are susceptible to low-power lasers in the presence of dyes, such as methylene blue, toluidine blue O, malachite green, and indocyanine green. aPDT uses these light-activated photosensitizer that is incorporated selectively by bacteria and absorbs a low-power laser/light with an appropriate wavelength to induce singlet oxygen and free radicals, which are toxic to bacteria. While this technique has been evaluated by many clinical studies, some systematic reviews and meta-analyses have reported controversial results about the benefits of aPDT for periodontal treatment. In the light of these previous reports, the aim of this review is to provide comprehensive information about aPDT and help extend knowledge of advanced laser therapy.

## 1. Introduction

**Figure 1:**
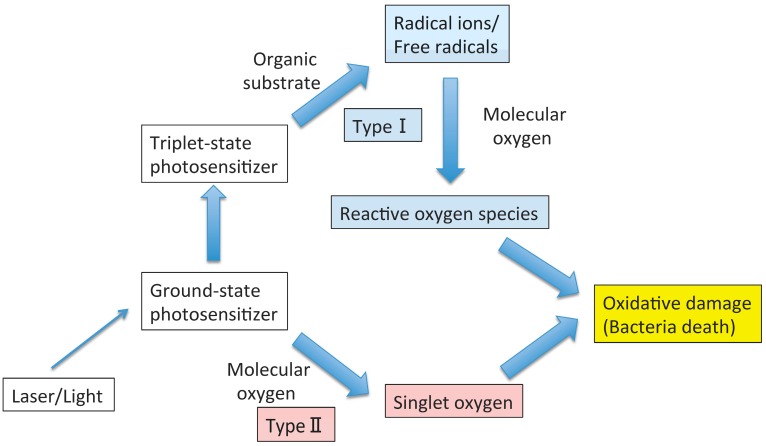
Photochemical mechanisms in photodynamic therapy.

Photodynamic therapy (PDT) utilizes singlet oxygen and free radicals produced by a light-activated photosensitizer to kill microbes. The photochemical process is initiated by a low-power laser/light at a relevant wavelength to excite the photosensitizer. The ground state photosensitizer absorbs light, resulting in a singlet state that can lose energy by fluorescence or undergo intersystem crossing to a triplet state with longevity. The latter state leads to a photochemical reaction that induces singlet oxygen, free radicals, and superoxide, which are cytotoxic, thereby inducing microbial killing [1] (Figure 1).

The initial application of PDT for selective toxicity was attempted more than a hundred years ago [2,3,4]. The discovery of antibiotics caused considerable stagnation for applications of PDT to infectious diseases in the 1940s. However, the recent rise in antibiotic resistance has shifted attention back to PDT. Interestingly, while PDT can eliminate antibiotic-resistant microbes [5], there is no information about microbes developing resistance to PDT [6].

Although an ancestor of PDT seemed initially and accidentally discovered for its antimicrobial effects [4], recent work on a practical PDT has focused on developing it as a cancer therapy [7,8]. The generated reactive oxygen species are toxic to cancer cells and lead to cellular death.

## 2. Antimicrobial PDT

Photosensitizers in antimicrobial PDT (aPDT), such as porphyrins, phthalocyanines, and phenothiazines (e.g., toluidine blue O and methylene blue), can target both Gram-positive and -negative bacteria by bearing a positive charge [9,10,11], suggesting that aPDT may be useful in oral applications, especially for periodontal treatment [12,13,14]. Activation of the photosensitizer can be induced by portable diode lasers that are cost effective compared with other types of lasers. Several studies have shown that periodontal bacteria are susceptible to PDT in planktonic cultures [15,16,17,18,19,20,21,22,23,24], plaque scrapings [23,25], and biofilms [23,24,26,27] using methylene blue [17,20,26], methylene blue-loaded polymeric nanoparticles [23], toluidine blue O [15,16,18,20,25,26], phthalocyanine [26,27], hematoporphyrin HCl [26], hematoporphyrin ester [26], a conjugate of poly-L-lysine and the photosensitizer chlorin e6 [19], indocyanine green [22], indocyanine green-loaded nanospheres [21], and safranine O [24] (Table 1). However, other studies have demonstrated incomplete destruction of oral pathogens [28,29,30,31,32].

**Table 1:** Photosensitizers used in antimicrobial photodynamic therapy (aPDT) for periodontal microbes.

Author and Year (Ref.)	Photosensitizer	Samples/Bacterial Strain	Conclusion
Bhatti *et al.*, 2002 [15]	Toluidine blue O	Planktonic culture/*Porphyromonas gingivalis*	Disruption of membrane functions associated with a decrease in membrane fluidity may contribute to the bactericidal effect of light-activated toluidine blue
Bhatti *et al.*, 1997 [16]	Toluidine blue O	Planktonic culture/*Porphyromonas gingivalis*	In the presence of toluidine blue O, a light dose-dependent increase in bacterial killing was attained (100% killing at 4.4 J)
Chan *et al.*, 2003 [17]	Methylene blue	Planktonic culture/*Actinobacillus actinomycetemcomitans*, *Fusobacterium nucleatum*, *Porphyromonas gingivalis*, *Prevotella intermedia*, and *Streptococcus sanguinis*	Using a diode laser of appropriate power and wavelength to deliver 60 s of irradiation could be a useful adjunct therapy with mechanical debridement for the prevention of re-colonization of subgingival lesions by pathogenic microorganisms
Matevski *et al.*, 2003 [18]	Toluidine blue O	Planktonic culture/*Porphyromonas gingivalis*	The data indicated that aPDT using a conventional light source was at least as effective as laser-mediated treatment *in vitro*
Souko *et al.*, 1998 [19]	A conjugate between poly-l-lysine and the photosensitizer chlorin e6	Planktonic culture/*Porphyromonas gingivalis* and *Actinomyces viscosus*	The cationic pL-ce6 conjugate may have applications in PDT of periodontal disease
Wilson *et al.*, 1993 [20]	Toluidine blue O; Methylene blue	Planktonic culture/*Porphyromonas gingivalis*, *Fusobacterium nucleatum*, and *Actinobacillus actinomycetemcomitans*	Low doses of light (22 J/cm^2^) were effective to kill bacteria *in vivo*, and the technique may be useful to eliminate periodontopathogenic bacteria from diseased sites
Nagahara *et al.*, 2013 [21]	Indocyanine green-loaded nanospheres	Planktonic culture/*Porphyromonas gingivalis*	ICG-Nano/c with low-level diode laser (0.5 W; 805 nm) irradiation might be useful as a potential photodynamic periodontal therapy
Topaloglu *et al.*, 2013 [22]	Indocyanine green	Planktonic culture/*Staphylococcus aureus* and *Pseudomonas aeruginosa*	The combination of ICG and 809-nm laser light was an effective antibacterial method to destroy antibiotic-resistant strains of Gram-positive and -negative bacteria
Klepac-Ceraj *et al.*, 2011 [23]	Methylene blue-loaded polymeric nanoparticles	Planktonic culture, plaque scraping, and biofilm/human dental plaque bacteria	Cationic methylene blue-loaded poly lactic-*co*-glycolic acid nanoparticles showed the potential to be used as carriers of methylene blue for photodynamic periodontal therapy Systems
Voos *et al.*, 2014 [24]	Safranine O	Planktonic culture and biofilms/*Streptococcus gordonii*, *Streptococcus mutans*, *Fusobacterium nucleatum*, *Aggregatibacter actinomycetemcomitans*, and *Porphyromonas gingivalis*	Oral pathogenic species in planktonic solution were suppressed significantly by antimicrobial photodynamic periodontal therapy with safranin O. Particularly for bacteria in a 24-h *ex vivo* biofilm, this method was more effective than treatment with 0.2% CHX. Both antibacterial treatments did not show any significant effect on the biofilm cultured for 72 h
Sarkar *et al.*, 1993 [25]	Toluidine blue O	Plaque scraping/human dental plaque bacteria	The use of low-power lasers, in conjunction with appropriate photosensitizers, may be a useful adjunct therapy to mechanical debridement for treating inflammatory periodontal diseases if similar effectiveness against subgingival plaque bacteria can be achieved *in vivo*
Dobson *et al.*, 1992 [26]	Methylene blue; Toluidine blue O; Phthalocyanine; Hematoporphyrin HCl; Hematoporphyrin ester	Biofilms/*Streptococcus sanguinis*, *Porphyromonas gingivalis*, *Fusobacterium nucleatum*, and *Actinobacillus actinomycetemcomitans*	Lethal photosensitization may be effective in eliminating periodontopathogenic bacteria from dental plaque
Wood *et al.*, 1999 [27]	Phthalocyanine	Biofilms/Human dental plaque bacteria	Confocal scanning laser microscopy of the biofilms showed that the photosensitizer was taken up into the biomass of the biofilm, and that significant cell death was caused by PDT

Toluidine blue O interacts with lipopolysaccharides present in the cell membrane of Gram-negative bacteria, even in the absence of light. However, upon exposure to a wavelength of 630 nm, it has maximal absorption and good photodynamic properties for killing various types of microbes *in vitro* [33]. Methylene blue shows maximal absorbance by exposure to a wavelength of 660 nm [17]. Indocyanine green has a high absorption at a wavelength of 805 nm by low-level diode laser irradiation [21].

Gram-negative bacteria are largely resistant to many photosensitizers used in aPDT [34]; however, some microbial species, such as oral black-pigmented bacteria, contain naturally occurring photosensitizers and are very susceptible to aPDT. It was demonstrated that the light band ranging from 380 to 520 nm induces a three-fold reduction in the growth of *Porphyromonas gingivalis*, *Prevotella intermedia*, *Prevotella nigrescens*, and *Prevotella melaninogenica* in dental plaque samples obtained from human subjects with chronic periodontitis [35]. Based on these findings, the same group proposed a phototherapeutic strategy by which daily exposure to visible light would gradually suppress the numbers of black-pigmented bacteria, leading to a shift of the microbial environment toward a healthy environment [36].

## 3. PDT as a Non-Antibiotic Antimicrobial Therapy

Generally, the metabolism of bacteria within a biofilm is notably more self-sufficient than that of planktonic cells. Furthermore, bacteria within a biofilm are better protected from harmful attacks. For example, oral-pathogenic species in a planktonic solution were significantly suppressed by either aPDT or chlorhexidine (CHX); however, in an *ex vivo* biofilm, aPDT was more effective at suppressing the bacteria than treatment with CHX [24]. Additionally, because of the low bacterial metabolic activity in the bulk layer of biofilm, lower amounts of drugs are absorbed and the reaction is weak. Because the surface layer of a biofilm also degenerates under the effects of antibacterial agents, further diffusion into the bulk layer is inhibited [37]. The reduced susceptibility of complex oral biofilms to aPDT may require the development of novel delivery and targeting approaches. Evolving therapeutic strategies for biofilm-related infections include the use of substances designed to target the biofilm matrix, non-proliferating bacteria within biofilms, and/or quorum-sensing bacteria [38]. The use of bacteriophages [39] and naturally occurring or synthetic antimicrobial peptides [40] may allow bacterial targeting without the emergence of resistance.

Currently, microbial viability recovery or any resistance mechanisms against aPDT has not yet been reported. Tavares *et al.* [41] performed a study investigating those issues and their results suggest that aPDT using Tri-Py^+^-Me-PF represents a promising approach to efficiently destroy bacteria since after a single treatment these microorganisms do not recover their viability and after ten generations of partially photosensitized cells neither of the bacteria develop resistance to the photodynamic process [41]. aPDT could rather be used to treat arthritis in mice caused by bioluminescent methicillin-resistant *Staphylococcus aureus* (MRSA) [42]. The advantages of targeted therapy are becoming more apparent, and the use of light alone, antibody- and bacteriophage-photosensitizer conjugates, and non-antibody-based targeting moieties, such as nanoparticles, are gaining increasing attention.

## 4. Periodontitis

Periodontitis is a common disease that causes tooth loss, and chronic inflammation induced by bacterial infection is the major cause of periodontium destruction [43]. Infection control by tooth surface debridement using hand instruments is a basic technique for therapy, and additional surgical procedures are sometimes needed. Systemic antimicrobial therapy is occasionally used for intractable disease. However, its side effects, including antibiotic resistance, should always be considered. Another option for antimicrobial therapy is local delivery to periodontal pockets. However, there are some disadvantages to this approach, such as the necessity for repeated treatments and its utility for small ranges of the periodontium. Additionally, the applied antimicrobial agents can cause decalcification and root surface softening [44].

It has been recognized that specific bacteria, rather than non-specific bacteria, are linked to chronic and aggressive periodontitis, which has led to the development of antimicrobial treatments for reducing these particular bacteria. It is difficult to visually identify the bacteria that are causing subgingival disease; thus, mechanical debridement solely cannot remove all sources of infection. Furthermore, dentinal tubes are opened by mechanical debridement. As a result, the remaining periodontal bacteria are able to penetrate into the dentinal tubes, and it is possible to confirm the formation of a biofilm shortly after treatment [45,46].

Some patients continue to show destruction of periodontal tissue after mechanical debridement. These patients often have risk factors, such as smoking, diabetes, hereditary factors, and systemic disease, which are accompanied by persistent infection with one or more specific periodontal pathogenic bacteria [47,48,49].

## 5. Periodontitis and Laser Treatment

Although the photosensitizers used in aPDT are generally activated by laser/light at specific wavelengths, sometimes the lasers used for periodontal treatment do not rely on the activation of a photosensitizer but on the interaction of the laser alone for the treatment of periodontal disease. There are several types of lasers utilized for periodontal treatment. CO_2_ lasers have mild root conditioning, detoxification, and bactericidal effects on contaminated root surfaces [50,51], but unfortunately, they also have the potential to produce thermal damage in the periodontal pocket and surrounding tissues [52]. The application of Nd:YAG and diode lasers similarly has bactericidal and detoxification effects, and this technique shows clinical benefits as an adjunct to nonsurgical periodontal therapy [53,54,55,56,57,58]. A diode laser is also utilized for low-level laser therapy, which is expected to promote healing by collagen synthesis and angiogenesis [59,60]. The better healing is observed when this is utilized for periodontal surgery [61,62]. The Er:YAG laser has the most clinically useful effects, including calculus and cementum bounding endotoxin removal, bactericide, and detoxification [63,64,65].

## 6. PDT as a Light Sterilization Therapy for Periodontitis

Bonito *et al.* showed that mechanical debridement cannot completely remove pathogenic bacteria [66]. Additionally, mechanical debridement alone only temporarily reduces the bacterial infection and may result in a return to pre-treatment levels in less than two weeks [45,46]. In the past decade, the limitations of conventional periodontal therapy have given rise to many attempts to introduce aPDT as an alternative treatment of chronic periodontitis [67,68,69,70]. aPDT has been confirmed to be effective as a non-antibiotic antimicrobial therapy [71,72].

Two main advantages are frequently cited for aPDT in comparison with other periodontal treatments. In aPDT, a photosensitizer placed directly into the pocket can be activated via an optical fiber also placed directly in the pocket, which helps to avoid damaging adjacent host tissue [73]. Additionally, the effects of aPDT are initiated by exposure to a light source, thus preventing the selection of resistant bacterial species [74]. Importantly, the eradication of biofilms and inactivation of inflammatory cytokines by aPDT has proven to be both effective and safe.

Human studies have produced contrasting results [12,69,75,76,77,78], and some systematic reviews have only partly discussed the adjunctive effect of aPDT [43,79]. A meta-analysis by Atieh [80] revealed supportive data for the potential improvements of using aPDT in conjunction with scaling and root planning (SRP) in periodontal treatment. That study found the association between those treatments is significantly related to a greater clinical attachment gain and a reduction in probing depth. Sgolastra *et al.* also conducted a systematic review indicating that the adjunctive use of aPDT and subgingival SRP can provide additional benefits in terms of reductions in pocket depth and gains in the clinical attachment level [12]. These benefits of combined treatment were observed only at the three-month follow-up time point. In contrast, no significant differences were observed at six months post-treatment. However, this finding is likely related to the small number of included studies that reported a follow-up time of six months. The most recent systematic review concluded that PDT with a diode laser adjunctive to SRP has beneficial effects with a moderate level of certainty (Table 2) [14].

**Table 2:** Systematic review of the adjunctive effects of antimicrobial photodynamic therapy (aPDT) for periodontal treatment (scaling root planing (SRP)).

Author and Year (Ref.)	Treatment Arms	Results
Smiley *et al.*, 2015 [14]	Test: SRP + aPDT; Control: SRP	aPDT with a diode laser adjunctive to SRP had a beneficial effect with a moderate level of certainty
Sgolastra *et al.*, 2013 [12]	Test: SRP + aPDT; Control: SRP	The use of adjunctive aPDT with conventional SRP provided short-term benefits
Sgolastra *et al.*, 2013 [79]	Test 1: SRP + aPDT; Test 2: aPDT; Control: SRP	The use of aPDT adjunctive to conventional treatment provided short-term benefits. There was no evidence of effectiveness for the use of aPDT as an alternative to SRP
Azarpazhooh *et al.*, 2010 [43]	Test 1: SRP + aPDT; Test 2: aPDT; Control: SRP	aPDT as an independent treatment or an adjunct therapy to SRP was not superior to SRP
Atieh *et al.*, 2010 [80]	Test: SRP + aPDT; Control: SRP	The use of aPDT in conjunction with SRP was associated with significant clinical parameter improvements

## 7. Animal Studies of the Effects of aPDT on Periodontitis

Favorable results with aPDT as an adjunct therapy to SRP have been reported in experimental rat periodontitis [67,68,81,82,83,84]. The progression of experimental periodontitis was substantially reduced by aPDT both radiographically and histologically [68]. Similar positive results were also obtained in furcation areas [81,82]. The rats treated with aPDT exhibited reduced numbers of tartrate-resistant acid-phosphatase-positive cells, weak immunoreactivity to the receptor activator nuclear factor-κB ligand, and strong osteoprotegerin immunoreactivity [83,84]. The efficacy of aPDT was also confirmed in periodontal infection of a beagle dog model [85,86]. Improvement in periodontal healing, associated with collagen organization, inflammatory cell infiltration, and bone loss, with the addition of aPDT has also been reported [87].

Experimental periodontitis models have revealed the microbiological and cytokine profiles resulting from aPDT [86]. A single application of aPDT, SRP, or the combination of both treatments all led to reductions in the levels of most bacterial species after one week [86]. However, increases in *P. intermedia*, *P. nigrescens*, and *T. forsythia* were observed following aPDT alone or in conjunction with SRP. After four weeks, regrowth of *P. gingivalis* and *T. denticola* were observed for all treatments, but a remarkable reduction of *A. actinomycetemcomitans* was observed following application of aPDT. Additionally, reductions in cytokine levels and bacterial numbers were observed regardless of the treatment used. The authors inferred that the different periodontal treatments tested had distinct mechanisms of action against the bacteria and, thus, might have additive or even synergistic effects [86].

## 8. Effects of PDT on the Host

A possible concern for the clinical application of PDT is the potential photocytotoxicity to host cells. However, it has been demonstrated that the doses of light needed for killing bacteria in PDT are much lower than those that are toxic for keratinocytes and fibroblasts [88]. In fact, some beneficial effects of PDT have even been reported in periodontal ligament cells, such as inhibition of inflammatory mediators, thus favoring cellular chemotaxis and the promotion of local vasodilation and angiogenesis [89]. In terms of the modulation of innate immunity, PDT acts on neutrophils and promotes their migration and integration [90]. PDT also inactivates host-derived cytokines, such as tumor necrosis factor-α and interleukin-1β, to inhibit E-selectin activation in endothelial cells [91]. PDT affects antigen-presenting cells, such as macrophages and Langerhans cells, by reducing their capacity to activate T-lymphocytes and weakens the inflammatory response [92]. Seguier *et al.* [92] also found that PDT targets different cell populations depending on the type of photosensitizer used in the treatment (e.g., liposomes and nanoemulsions). Fujimura *et al.* reported the effects of PDT on epithelial cells by irradiation with a low-level diode laser and an indocyanine green-loaded nanosphere coated with chitosan (ICG-Nano/c) [93]. The migration of epithelial cells and expression of Del-1 were significantly increased by diode laser irradiation with or without ICG-Nano/c compared with those in the control. These results suggest that low-level diode laser irradiation with or without ICG-Nano/c can suppress excessive inflammatory responses via these mechanisms in addition to its antimicrobial effect.

## 9. Effect of aPDT on Periodontitis with an Unusual Host Response

Some studies on the effects of aPDT in patients with aggressive periodontitis [94,95,96,97,98] have reported favorable results for the state of human subgingival flora. It has been suggested that both aPDT and SRP might be beneficial for nonsurgical treatment of aggressive periodontitis [94]. The most recent report from Moreira *et al.* showed additional clinical, microbiological, and immunological benefits of aPDT in patients with aggressive periodontitis [97].

Under specific periodontal conditions, aPDT can be a useful tool for antimicrobial treatment when conventional SRP is not effective [67] in medically compromised patients [67,99,100], children, and disabled people [27]. It has also been suggested that aPDT may be useful as an adjunct therapy to SRP for persistent periodontitis that is strongly related to the presence of *P. gingivalis* and *P. intermedia* [101]. aPDT counteracts the impaired healing in diabetic and/or immunosuppressed animals [102,103,104]. Additionally, aPDT has been shown to reduce bone loss and promote the repair of bone tissue, which is modulated by immunosuppressive drugs [105,106,107,108]. The influence of aPDT on periodontal bone loss related to diabetes in rats was initially reported by Almeida *et al.* [67]. Their histometric data showed that aPDT using toluidine blue O and a GaAlA laser produced less bone loss compared with rats treated only with scaling root planing or toluidine blue O in both diabetic and non-diabetic rats by increasing the diffusion of oxygen through the tissue. This effect favored the repair process because collagen production by fibroblasts in the extracellular space occurs only in the presence of high rates of oxygen pressure.

## 10. Conclusions

While many preferable outcomes *in vitro* and *in vivo* have been demonstrated for the use of aPDT, there is some variability in the results of this technique in clinical practice. However, the majority of systematic reviews conclude that the inclusion of aPDT as an adjunct to nonsurgical periodontal treatment seems to be therapeutically useful. Further studies of aPDT are needed for establishing this as a beneficial adjunct treatment for periodontitis.
